# The State Treatment of Venereal Diseases: The New Regulations

**Published:** 1916-08-26

**Authors:** 


### THE STATE TREATMENT OF VENEREAL DISEASES.

### The New Regulations.

Speedy action has been taken to put into effect the recommendations contained in the Final Report of the Royal Commission on Venereal Diseases. His Majesty's Government, having decided that it is important in the national interest that immediate steps should be taken to make provision for the diagnosis and treatment of these diseases, the Local Government Board has issued circulars and made regulations dealing with the subject. These will mark an epoch in the vicissitudes of legislation concerning, and of treatment of, these affections.

The duty of initiating a new crusade against venereal disease is to be entrusted to the County and County Borough Councils, and, in the case of London, the City Council. Each of these bodies is under the regulations, which it is unnecessary for us to quote *in extenso*, required to prepare a scheme to be submitted for approval to the Local Government Board. The arrangements to be made in order to provide free skilled treatment and adequate laboratory facilities for diagnosis are to consist, in the first instance, of agreements with and the payment of subsidies to those existing hospitals which are able and willing to carry out the work.

The trend of modern public health administration towards co-operation with curative medicine is once again exemplified, the aim and object of the regulations being clearly twofold:—(i) to provide for early diagnosis and treatment, with a view to (ii) the prevention of the spread of certain communicable diseases. These are defined as: (*a*) syphilis, (*b*) gonorrhœa, and (*c*) soft chancre. It is probable that questions will arise as to the inclusion for treatment of conditions other than acute or communicable and of complications; it may be well, therefore, to point out that "the special facilities to be provided should be designed chiefly for cases in which the diseases are in the communicable stage"; in fact as regards syphilis, the primary and secondary stages of the disease, and not tertiary or parasyphilitic manifestations, are referred to.

The organisation is to include:—

A. The establishment of clinics at hospitals or elsewhere.

B. The supply of salvarsan or its substitutes, under specified conditions; and

C. The provision of laboratory facilities for diagnosis and for guidance in treatment.

A. Clinics.—The intention is that in each unit or area (County or County Borough) hospital treatment shall be provided for patients coming from the area, preferably at hospitals to which a medical school is attached. The clinic should be available free, "to all comers without distinction as to means or as to place of residence." It would appear that patients may elect to apply for treatment at an institution in an area other than that in which they reside, or at one with which the Council of their area has made no arrangements for treatment. A hospital committee may, however, agree with local authorities for the treatment of patients coming from more than one administrative area. Patients' home addresses are to be kept confidentially for reference, but never communicated to others; case-records and reports will be identified by letters or number. Treatment will be, for the most part, carried out at out-patient clinics, some of which should be held in the evening and which should not be labelled as specially for venereal diseases or made to distinguish patients attending them. It is intended that provision shall be made, either at the hospital to which the clinic is attached or elsewhere, for the use of a small number of hospital beds, available under conditions which will not involve the identification of the specific disease by other patients.

B. Salvarsan or its Substitutes.—It is proposed that for the present these preparations shall be supplied gratuitously only if administered by the medical officer of a clinic or by a medical practitioner, authorised by him, who is known to have had experience in such administration. Arrangements for administration by the medical officer, in appropriate cases, at the patient's home may be made.

C. Laboratory Facilities.—It is intended that existing large pathological laboratories, such as those attached to the general hospitals, universities, or medical schools, be utilised. The examinations required will, of course, include the detection of the *treponema pallidum* and Wassermann tests. The recommendation in favour of large pathological laboratories is made especially on account of the advantages in efficiency and economy of making Wassermann tests at a laboratory in which a large number of specimens are examined, and of employing a pathologist skilled in this special work. Whilst it is not essential that the laboratory should be situated in the administrative area which it serves, there are distinct and obvious advantages in having the clinic and the laboratory, whenever possible, upon the same premises, and the medical officer of the clinic should be in close communication with the pathologist, who should be personally responsible for his share of the work and should sign all laboratory reports.

Whilst it is believed that "at the present time a relatively small proportion of patients suffering from venereal disease go for treatment to their own doctors," every endeavour is to be made to encourage full co-operation and consultation between the general practitioner and the medical officer of the clinic. The clinics are to be open for the attendance of practitioners and medical students. Facilities are to be given for the gratuitous examination at the laboratories of material obtained by the practitioner, for instruction in the methods of obtaining such material, and for the supply of the necessary apparatus.

"No short-sighted parsimony" is to be allowed to mar the benefits of these schemes. Of the expenditure incurred by the Councils under arrangements approved by the Local Government

Board, the Board will repay 75 per cent., whilst it is stipulated that the hospital authorities concerned in carrying out these arrangements shall not have their administration interfered with, and shall, if they so desire, have defrayed out of public funds the whole of the extra expenditure incurred by them. Certain reports and statistics will have to be furnished by their officers. Laboratory facilities and the means for obtaining salvarsan and its substitutes are to be available to Boards of Guardians and their medical officers; 75 per cent. of approved expenditure will be repaid.

Such (very briefly outlined) is the framework upon which consultation is now to take place, and agreements are to be entered into, between the authorities concerned, with a view to placing "within reach of the population" those modern methods of diagnosis and treatment of venereal diseases which the Royal Commission considers are not now generally available.

